# What adults with ADHD want to know: A Delphi consensus study on the psychoeducational needs of experts by experience

**DOI:** 10.1111/hex.13592

**Published:** 2022-08-23

**Authors:** Christina Seery, Margo Wrigley, Fiona O'Riordan, Ken Kilbride, Jessica Bramham

**Affiliations:** ^1^ UCD School of Psychology University College Dublin Dublin Ireland; ^2^ National Clinical Programme for ADHD in Adults Health Service Executive Dublin Ireland; ^3^ ADHD Ireland Dublin Ireland

**Keywords:** adult ADHD, attention‐deficit/hyperactivity disorder, Delphi method, digital mental health intervention, experts by experience, psychoeducation

## Abstract

**Introduction:**

A lack of knowledge about attention‐deficit/hyperactivity disorder (ADHD) can contribute to feelings of distress and difficulty in seeking and accepting an ADHD diagnosis. The present study uses a Delphi consensus design to investigate the psychoeducational needs of adults with ADHD and the information about ADHD they would like included in digital health interventions for adults with ADHD. Inclusion of perspectives of service users in developing such interventions ensures that they are evidence based and addresses the risks of engagement barriers.

**Methods:**

The expert panel consisted of 43 adults with ADHD (age range: 23–67 years). Panel members were asked to rate the importance of the proposed topics and provide additional suggestions. Suggested topics and topics that did not achieve consensus were included for ranking in the second round.

**Results:**

Interquartile ratings were used to determine consensus. A high consensus was achieved in both rounds, with an agreement on 94% of topics in the first round and 98% in the second round. Most topics were rated as important or essential.

**Conclusions:**

The findings highlighted that adults with ADHD want to learn about many different aspects of ADHD and the importance of considering their perspectives when developing psychosocial interventions. Findings can be applied when creating psychoeducational content for adult ADHD.

**Patient or Public Contribution:**

Adults with ADHD were recruited to the Delphi panel to use an experts‐by‐experience approach. In doing so, we are engaging service users in the development of a psychoeducational smartphone app. The evaluation of the app will involve interviews with app users. Additionally, the present study was developed and conducted with ADHD Ireland, a charity based in Ireland that advocates for people with ADHD.

## 1 INTRODUCTION

Attention‐deficit/hyperactivity disorder (ADHD) has been increasingly recognized as a lifespan disorder, with 21%–38.8% of those diagnosed as children fulfilling the diagnostic criteria as adults.^1^, ^2^ Psychoeducation is a standard component of psychosocial treatments for ADHD.^3^ Multiple studies have shown the benefits of group‐based psychoeducation for adults with ADHD, demonstrating improvements in knowledge of ADHD, self‐esteem, psychological well‐being and ADHD symptoms.^4^, ^5^, ^6^ The research has focused mainly on the effectiveness of group‐based psychoeducation. As clinics for ADHD are often busy services with high demand,^7^, ^8^, ^9^ psychoeducation provided by digital health interventions may be helpful and an opportunity for patients to engage with psychoeducational content outside of the clinic, reducing the burden on services.

Digital health interventions, such as smartphone apps, have risen in popularity in recent years,^10^ with research demonstrating their effectiveness for various mental health conditions.^11^, ^12^, ^13^ The guidelines for ADHD provided by the National Institute for Health and Care Excellence advise clinicians to recommend helpful supports, like apps, to patients to facilitate adherence to treatment.^14^ Apps can be useful for adults with ADHD, providing them with psychoeducation,^15^ digital diaries and reminder notes at low or no cost.^16^ Preliminary randomized studies demonstrate the potential effectiveness of apps for ADHD. An intervention using mobile apps to support organizational skills has been shown to significantly decrease inattention and hyperactivity symptoms and depression scores in adults with ADHD.^17^ Additionally, a chatbot mobile app appeared to improve symptoms of attention deficit and emotional lability. Greater engagement with psychoeducational materials within the chatbot was associated with lower ratings of symptoms,^18^ which may suggest a supportive role of psychoeducational digital health interventions for adults with ADHD. In children and young people with ADHD, digital health technologies may help support self‐management.^19^ A digital health intervention aimed at improving attention and related cognitive control processes significantly improved objective scores of attention, ratings of ADHD symptoms and functional impairment.^20^ Feasibility and pilot studies demonstrated the utility of an iPad app for monitoring the behaviour of children with ADHD in their classrooms.^21^

In addition to effectiveness studies, qualitative research appears to demonstrate a willingness to engage with digital health interventions for ADHD. Many adults with ADHD learn to rely on external resources like apps to help support their organizational skills.^22^ People with ADHD and healthcare clinicians also frequently use websites for information on ADHD but want access to trustworthy and reputable resources. They recognize the potential supportive role of remote monitoring technology. The ideal app would be reliable and trustworthy and would include features for monitoring and tracking side effects and symptoms.^23^ Furthermore, Flobak et al.^24^ used a participatory design process to include the input of adults with ADHD in the development of their psychoeducational online therapeutic content. Some participants in their clinical trial described the codesigned videos as the most positive aspect of the intervention. Through these videos, participants recognized and related to the depicted challenges, characters and situations and viewed the video protagonists as companions and role models for change. A systematic review of ADHD apps downloadable from the app store found that 109 apps were available catering to a wide variety of users, with 30% targeted towards adults with ADHD. However, very few of the apps described their evidence‐based support, and only two of the apps were being subjected to empirical evaluation.^25^

The challenge in delivering digital health interventions is the high attrition rate of users despite those users' initial willingness to utilize such interventions. For example, Hidalgo‐Mazzei et al.^26^ found that only 30% of their original 201 participants continued to use the app for people with bipolar disorder after 6 months. Similarly, there was a significant reduction of check‐ins and total usage time of Jang et al.'s^18^ mobile chatbot for ADHD in the second and third weeks of the study. There were no significant differences between the chatbot and control conditions in acceptability, potentially because of the chatbot's interface. Additionally, a qualitative study with clinicians and children and young people with ADHD found that the characteristics of the top 10 apps in the marketplace did not match the participants' views of what was important and helpful to include.^27^ It may be that while there is an initial interest in the digital health interventions offered, the actual experience of them is negative due to lack of input from people with ADHD at a developmental stage. A review of technology research for ADHD conducted by researchers with ADHD found that technology development and research teams neglected the views of people with ADHD, which can lead to resistance towards the intervention. The focus of technologies has often been on disciplining or suppressing ADHD traits. Researchers and developers in digital health interventions for ADHD should aim to centre ADHD voices.^28^ Engaging service users in the design of digital health interventions is also essential to meeting their needs and to ensuring that the technologies are more engaging, feasible, acceptable and effective.^29^, ^30^

While service user perspectives are crucial for the successful implementation and adoption of digital health interventions, there is no research on the specific educational needs of adults with ADHD to inform their development. Such research offers valuable insight into the target users' concerns and desires, which can help to address barriers to use and centre the ADHD voice. One way to do so is to understand the priorities of adults with ADHD in the content of digital health interventions. Delphi methods can be used to aggregate ideas, make future predictions, determine expert opinions and achieve consensus by conducting a series of ‘rounds’ of questionnaires with experts.^31^, ^32^ Achievement of consensus is valuable for the development of digital health interventions as ascertainment of a breadth of needs allows for the intervention to potentially resonate with a wider range of service users. The Delphi method has been commonly used in health research,^33^, ^34^, ^35^, ^36^, ^37^ with a select few in the ADHD literature,^16^, ^38^, ^39^, ^40^ and to identify the best forms of personalization and monitoring indicators in a digital health intervention for grief.^41^ While studies traditionally include a panel of professional experts, with many including subpanels with patients, the Delphi method with experts by experience may potentially inform the development of interventions. They are also beneficial as service users' priorities may differ from those of professionals.^42^ For example, Novais et al.^43^ conducted a modified Delphi method involving caregivers of people with neurocognitive diseases to identify a consensus of caregivers' common needs. This expert‐by‐experience methodology has also been applied with children to identify what they feel is essential in an intervention on self‐concept,^44^ to explore adolescents' needs following nonconsensual sexual images^45^ and to define autistic burnout and highlight the specific needs of autistic adults.^46^

A consensus of expert‐by‐experience views allows researchers to understand the priorities that service users have for the features and content of interventions. Identifying the priorities of service users and hierarchizing them can help in the design of targeted interventions. Understanding of the consensus of priorities enables the intervention to address the needs of as many people as possible.^43^ Identifying priorities for neurodivergent people is particularly pertinent as research has shown that there are gaps between available and desired interventions for neurodivergent people and that outcomes are not aligned with the needs of the community.^47^ In ADHD digital interventions, the focus has traditionally been on disciplining ADHD traits^28^ and adopting a medical model of disability, thus marginalizing neurodivergent users.^48^

A Delphi method aimed at achieving consensus on educational topics and adaptions to interfaces between experts by experience could help identify priorities of adults with ADHD. By identifying and achieving consensus in priorities of adults with ADHD, researchers and developers can ensure that they are listening to ADHD voices and addressing the breadth of needs from digital health interventions. Therefore, a Delphi method of achieving consensus between experts by experience may produce a helpful ranking of educational topics that can inform digital health and psychosocial interventions for adults with ADHD.

### 1.1 The present study

The current study is the initial step in a project to develop smartphone‐based psychoeducation and a virtual workshop series for adults with ADHD. The app will be specific to Ireland and developed for the Health Service Executive's (HSE's) National Clinical Programme for Adults with ADHD with ADHD Ireland. As digital‐based interventions have high rates of attrition,^26^ the present study was conducted to include the perspectives of adults with ADHD in the development of the app. By using a Delphi consensus method with experts by experience, the specific aims of this study were (1) to identify and prioritize what adults with ADHD want to know about their condition to develop appropriate psychoeducational content and (2) to identify and prioritize what adaptations adults with ADHD want in the presentation of the app material (e.g., text size, videos, audio clips).

## 2 MATERIALS AND METHODS

### 2.1 Delphi methodology

In the present study, a modified Delphi method was used to collect and merge the opinions of adults with ADHD. Participants receive controlled feedback based on their responses in subsequent rounds. They are informed of their response or score on the Likert scale and the average response of the other panel members and then allowed to revise their choices,^49^ which supports the convergence towards consensus. A modified Delphi method was used, which presented participants with a predeveloped list of potential topics and adaptions for a digital health intervention and the opportunity to supply additional suggestions through an open‐ended survey. A modified Delphi method was chosen in an effort to reduce participant burden so that participants did not feel that they had to suggest a wide variety of topics and adaptions and could instead provide additional ones or respond to the topics and adaptions presented. This study received ethical approval from the first and senior authors' university ethics committee.

### 2.2 Topics development

The list of topics for the first round of the study was developed similarly to Ahmed et al.^38^ and Langbecker et al.^50^ by producing a list before the first round using thematic analysis to reduce participant burden. Content was derived from five national nonprofit organizations for ADHD as they each aim to raise awareness of ADHD and provide information about ADHD in an open and accessible way, similarly to how our future digital health intervention will: AADD‐UK, ADHD Ireland, ADHD New Zealand, the Centre for ADHD Awareness Canada and Children and Adults with ADHD (based in the United States). Organizations were selected for inclusion if their countries of origin were English‐speaking, their organizational aims included providing information about ADHD and they had content specifically for adults with ADHD. Webpages for adult ADHD and general knowledge about ADHD were included for thematic analysis.

#### 2.2.1 Thematic analysis

In line with Braun and Clarke's^51^ recommendations, the first author familiarized herself with the data by initially reading through the organization's website content and rereading content when it was selected for inclusion. Initial codes were generated and regularly reviewed with the senior author. Following initial coding, the first author searched for themes in the codes before reviewing them. Themes were defined and named. A thematic map was developed.

Overall, four main themes were identified: background information on adult ADHD, diagnosing ADHD, interventions for ADHD and living with ADHD. Five subthemes were observed within the theme of living with ADHD: relationships, laws and rights, occupational and educational settings, finances and driving. The themes formed the categories of topics that could be presented in the smartphone app. Potential topics were derived from codes in the themes for 119 topics.

### 2.3 Participants and recruitment

Delphi studies do not require large samples, as the reliability and validity of achieving consensus appear to be unaffected by sample size.^49^ Previous Delphi studies on ADHD have had final sample sizes that ranged from 21 to 58.^38^, ^39^, ^40^ Adults who self‐identified as having ADHD were recruited through ADHD Ireland's mailing list and Twitter with ADHD hashtags. Overall, 25 females, 17 males and 1 nonbinary person with ADHD completed the first round (age range: 23–67, *M* = 39.7, SD = 9.5).

Participants who provided their email addresses and consented to be contacted for the next round of the study were invited to complete the final survey. A total of 26 participants completed the second round of the survey (attrition rate of 40%), with 15 not following the link to the second round and two dropping out of the study before reading the information sheet.

### 2.4 Materials and procedure

Participants read the information sheet that described the nature of the study and proceeded to the Likert‐type scale after providing full consent. Participants were asked whether the proposed 119 topics should be included in a psychoeducational app by ranking them according to the following scale: ‘should not be included’, ‘unimportant’, ‘unsure’, ‘important’ or ‘essential’. Following rating of the topics, an open‐ended question asked participants to include any other topics they felt should be added.

Participants were then asked to indicate a preference for how the app content is displayed on a scale of ‘extremely unhelpful’, ‘unhelpful’, ‘unsure’, ‘helpful’ or ‘extremely helpful’, followed by open‐ended questions asking if there were adaptions to the presentation of topics they believed would be beneficial and if they had other suggestions for the presentation of app content. Participants were invited to provide their email addresses should they wish to be contacted for the second round of the survey, and they were asked to create a unique identifier code to identify their responses.

The first round of responses was analysed. A shortened list of potential topics was derived from the responses and included for the second round. Following analysis, individual surveys were developed for the participants, including their former responses and the group's average. Participants were contacted with personal links and invited to participate in the second and final round of the study. The shortened list of five topics, including the additional suggestions by participants from the first round (*n* = 37), was presented with the same scale. Suggested topics were excluded if they were specific advice for the app (e.g., recommendations of other apps) or personal comments. Following data collection, responses were analysed.

### 2.5 Data analysis

The consensus was determined by using interquartile ranges (IQRs). IQRs are an objective measure of observing agreement between panel members by measuring variance.^52^ On a Likert scale with four or five options, an IQR of 1 or less is considered a high level of consensus.^53^, ^54^ An IQR of 1 or less demonstrates that respondents showed slight variance in their responses, and as such, there is a strong consensus. IQRs are calculated by subtracting the lower median from the upper median of scores. Topics with an IQR of more than 1 were included in the second round.

## 3 RESULTS

A total of 156 topics were presented to the panel members between the two rounds of the study, including the original 119 topics developed from the thematic analysis of ADHD organization websites and the additional 37 topics that participants suggested. The complete list of topics can be found in the online Supporting Information Material.

### 3.1 The first round

In the first round of the study, consensus was achieved for 94% of the 119 topics, with 112 having an IQR of 0–1. Topics with an IQR of 0 were as follows: the prevalence of ADHD (mostly rated as important), executive functioning (essential), inattention (essential), the prevalence of specific learning disabilities (important), available intervention options (essential), staying focused (essential), managing procrastination (essential), time and task management (essential) and improving organization (essential).

Topics that did not achieve consensus (IQR of 2) were the history of ADHD, the gender ratio of ADHD, transitioning from children and adolescent mental health services to adult services, occupational therapy, recognizing snake oil treatments, notifying the Road Safety Authority (RSA), driving insurance and weight management.

Most of the topics (75) were rated as essential. No topics were rated lower than ‘unsure’ (four topics in total).

The suggestions for presentations of app content all achieved a high consensus of 1. Only graphics were considered essential, while text‐based information and videos were important. Participants were unsure about the utility of audio clips. The most frequent suggestion for the adaption of content was to keep videos as short as possible:

Videos under 3 min. Videos must include Cc or transcript below the video.

Synthetic videos less than 2 min; efficient, accessible… … less overwhelming with link to or longer videos behind….

Quick ‘information pills’ videos with infographics will be a great way of explaining complex information….

Other insightful suggestions were that content should be attention‐grabbing, colourful and exciting and that it should use graphics to communicate. Participants recommended that text‐based content should have bigger font and use bullet points, with one participant suggesting a Powerpoint‐style approach to graphics and bulleted text: ‘PowerPoint type with graphics & billeted [sic] text, short & snappy, an ADHD'er won't read long paragraphs, should be colourful & interesting & really focus on the benefits of ADHD’.,

Additional suggestions for the presentation involved gamification of the content with a reward‐based system. One user stated the following:

With ADD, you're caught between the horns of a short attention span, hyperfocus, distractibility and easy frustration. Influenced by transient variables. So for content, offer variety. A simple example is to have an optional (well) recorded voice for a long read, like a podcast with a transcript. Or, if gamifying an infographic, pop up an option for haptic feedback. Create positivity wins for people who need them—get user to create a personal playlist of favourite music/recordings of family/displays an image that plays in full when something is achieved—a walk in the park, a burdensome chore, an early night's sleep….

A bookmark feature was also recommended by another participant, as well as a timestamp feature for long videos or audio clips.

### 3.2 The second round

The second round included five topics from the initial round that did not achieve consensus and topics suggested by participants. A high agreement was reached on almost all 42 topics in the second round, except ‘gender ratio’ (with an IQR of 2 and an average of ‘unsure’, remaining unchanged from the first round). Items with an IQR of 0 included comparing ADHD functioning with more typical functioning as a way of recognizing ADHD (important), stigmatizing responses to ADHD (important), disassociation (important), risk management (important), pros and cons of hyperfocus (important) and disconnecting from hyperfocus (important). The only topics rated as ‘essential’ in the second round were recognizing abusive romantic relationships, up‐to‐date research on ADHD and what managed ADHD looks like.

## 4 DISCUSSION

The present study aimed to identify and prioritize the psychoeducational content that adults with ADHD want to know and whether the content should be adapted in a particular way to best develop a digital health intervention that will meet the needs of its service users. To do so, 119 potential educational topics were developed by analysing ADHD organization websites and conducting a modified Delphi study comprising two rounds. Overall, agreement was achieved on 94% of the items in the first round, and only the ‘gender ratio’ did not reach a complete consensus by the end of data collection. To the best of the author's knowledge, this is the first study to provide insight into the priorities of adults with ADHD for informational needs from a digital health intervention and to use a Delphi methodology utilizing experts by experience of ADHD rather than professionals with academic or clinical knowledge of ADHD.

Both rounds observed a very high consensus in participants' ratings, with most topics being rated as important or essential on average. It may be that adults with ADHD value a breadth of information and education about many different aspects of their ADHD. After a diagnosis of ADHD in adulthood, Aoki et al.^55^ found that many participants are highly interested in learning about their ADHD and seek out information in pamphlets, books and online. It may be that participants in the current study felt that a significant number of the topics would be of value in a digital health intervention and, therefore, rated many as important or essential. Alternatively, the high acceptance rate may reflect the methodology of the study. In their Delphi study, Ahmed et al.^38^ observed similarly high consensus and acceptance rates, which resulted in a prompt question list for parents of children with ADHD. Like Ahmed et al.,^38^ the present study used thematic analysis to create the initial list of topics to reduce participant burden. The thematic analysis was conducted on websites that provided information about adult ADHD. The high acceptance rates may indicate that the websites were providing information that met the needs of adults with ADHD. A third possibility for the significant number of topics that were rated as important or essential could be that the panel struggled to discriminate between the topics and what the content would have covered. For example, participants may not have understood ‘executive function’ or were unsure what the information on executive function would detail. This may have led to panellists being more inclusive in their ratings, as it might have been challenging to be discerning.

Of the 43 panellists who completed the first round, 26 participated in the second round. Other Delphi studies with the expert‐by‐experience methodology have demonstrated wide variation in participant dropout, with attrition rates of 16%,^43^ 22%^46^ and 34%.^42^ A systematic review of Delphi studies observed a higher level of round two responses when panellists were recruited through treatment centres.^56^ As participants for the present study were recruited through ADHD Ireland and social media, attrition could have possibly been reduced by advertising the research in clinical settings. However, the research was conducted during Autumn 2020, during COVID‐19 lockdowns in Ireland, and it likely would not have been possible to recruit in clinical settings. Alternatively, the attrition rate of 40% may have been due to the time lapse between rounds, as participants were recruited to the first round in October 2021 and the personal links for the second round were emailed in mid‐December. The delay between rounds may have led to participants forgetting about or losing interest in the study. It may also be that participants felt that the first round of the study required too much time, which can lead to dropout in Delphi studies.^57^ The number of items in Delphi rounds is also associated with dropout, with more items leading to increased rates of attrition.^58^ Participants may have understood there to be a similar number of items (over 100) and open‐ended questions in the second round, and this could have contributed to 17 participants dropping out.

Items rated as essential reflected participants' desire to understand their ADHD in‐depth (e.g., its nature, executive functioning, impulsivity and inattention). Aoki et al.^55^ observed that following a mixture of confusion and self‐stigma after being diagnosed, Japanese adults started seeking out information on ADHD as a disorder to learn how to cope and share with their loved ones. Clinicians or digital health interventions for adults with ADHD should aim to provide adults with a comprehensive (but not overwhelming) overview of ADHD while emphasizing the advantages of ADHD. Considering how to best adapt content might be particularly relevant when providing an overview of ADHD, as this will likely be the earliest discussion with someone who was recently diagnosed. The use of graphics was rated as essential for the app. Clinicians may also want to use pictures or visuals when explaining ADHD, particularly for complicated concepts like executive functioning.

Mobile apps or digital health interventions can help address gaps in service provision due to a global shortage of clinicians and access to care in rural areas.^59^ Apps are more likely to reach vulnerable patient groups than other digital health interventions^60^ and also have the potential to engage service users in their care, increase access to and use of evidence‐based interventions and provide supports after formal treatment has concluded.^61^ The anonymous nature of apps may also help to protect the user from experiences of stigma.^62^, ^63^ Additionally, from a service perspective, digital health interventions and apps may be a cost‐effective resource,^64^, ^65^ although research is still limited. Results from the present study have been used to inform the content of a psychoeducational app. This app will offer evidence‐based psychoeducation and self‐help techniques to adults with ADHD in Ireland, many of whom are likely waiting on services as the HSE's National Clinical Programme for ADHD in Adults gradually launches ADHD services around the country.^66^ The app will hopefully provide a helpful resource to adults with ADHD, with content addressing the topics ranked as essential in the present study.

By drawing on the present study's findings in the development of digital health interventions, researchers and clinicians can ensure that they are meeting the informational priorities of adults with ADHD. This may help to reduce attrition rates and usability challenges^18^, ^26^ and provide apps that are actually meeting the users' needs.^27^ Researchers and clinicians should also look to continue to draw on the perspectives of adults with ADHD during other stages of development to ensure that service users identify and resonate with the content^24^ and that the interface is accessible.^18^ Developers can draw on the present study's findings on how contents should be presented on the apps to help design the interface. Clinicians may also want to consider findings during postdiagnosis discussions or follow‐up appointments with recently diagnosed patients. Results could also inform any psychoeducational content, such as booklets or information on websites, that clinicians direct patients towards.

### 4.1 Limitations

Findings should be considered with awareness of the study's methodological limitations. The Delphi method aims to produce a consensus of experts' priorities.^31^ The present research wished to identify and prioritize the educational needs of adults with ADHD from a digital health intervention, similar to other studies that have highlighted the priorities of adults with psychosis^42^ and caregivers of people with neurocognitive diseases.^43^ However, this methodology does not allow for exploring experience. Future research that uses traditional qualitative methods will be essential to investigating what adults with ADHD would like to learn about their ADHD and the materials or interventions they received after a diagnosis, similar to Cogley et al.'s^67^ study on the information needs of spinal cord‐injured patients and their family members. This will also offer additional insight into the therapeutic and emotional impact of learning about ADHD for adults.

The study was advertised as recruiting adults with ADHD and required those who identified as such to self‐select into the research. However, participants did not provide evidence of a clinical diagnosis. This was to facilitate participation from adults who self‐identify as having ADHD but who may not yet have a diagnosis or opted not to pursue a formal diagnosis, due to significant waiting lists and barriers to ADHD care.^68^, ^69^, ^70^ Research has indicated that college students who self‐identify as having ADHD but are undiagnosed demonstrate more neuropsychological impairment than non‐ADHD peers,^71^ which may possibly support the validity of self‐diagnosis. However, we did not collect data on which participants self‐diagnosed and were formally diagnosed and could not analyse any significant differences between these groups or their frequencies. Not collecting these data or requiring evidence of clinical diagnosis may have led to a recruitment bias. Future research could explore differences and similarities in educational and psychological needs between adults with formally diagnosed and self‐identified ADHD.

Another limitation of the research is a lack of a stability criterion. While Delphi studies generally focus on consensus, stability between rounds is provided by the consistency of responses between rounds.^72^ The findings of systematic reviews on Delphi techniques have shown that the stability of judgements does not typically play a central role in Delphi articles.^32^ The 112 items that achieved consensus in the first round of the present study were not presented in the second round to reduce participant burden and to minimize attrition. However, had these been presented to participants, it would have been possible to assess the stability of responses. Similarly, we did not analyse disagreement or diversity of needs and perspectives across users. As the aim of the study was to identify the priorities of most participants, and therefore hopefully meet the needs of many of the app users, the focus of the research was on achieving consensus. However, disagreement between panellists is often a valuable and insightful outcome.^73^

The original 119 topics were derived from the thematic analysis of the content available on the websites of multiple ADHD organizations. The language of these topics was drawn directly from the websites. However, some of the language may have been overly medical (e.g., comorbidities, transitioning from CAMHS to adult services). This may have caused participants confusion and led to rating the topic as more important due to a lack of understanding. Technical language may have also led to an educated participant sample with a high literacy level. As the level of education was not asked in the demographic questions, this potential effect cannot be fully considered.

Eight topics did not achieve consensus in the first round. However, three were not included in the list presented during the second round. Two of these topics referred to notifying the RSA and driving insurers about ADHD. Information on these topics was provided on two ADHD organizations' websites. It was later discovered that informing the RSA is not necessary in Ireland, and to minimize confusion, the item around insurance was also removed. Weight management was not included to avoid redundancy, as nutrition and exercise were both rated as essential on average.

## 5 CONCLUSION

The present modified Delphi study aimed to determine what adults with ADHD want to know about ADHD from a psychoeducational smartphone app. Our findings highlight that adults want to learn about many aspects of ADHD, ranging from the condition itself to how it can affect their daily lives and techniques to manage it. A high consensus was achieved in both rounds, with most topics rated as important or essential. The high rankings of topics also underline the importance of providing psychoeducation to adults with ADHD. Findings will be used to inform the development of a psychoeducational app and can also be applied to content for interventions or as a reference point when discussing ADHD with someone, mainly if they have been recently diagnosed.

## AUTHOR CONTRIBUTIONS

Christina Seery contributed to the research design, data collection, data analysis, interpretation of results and writing. Ken Kilbride contributed to the research design, data collection and editing. Margo Wrigley and Fiona O'Riordan contributed to the research design, interpretation of results and editing. Jessica Bramham contributed to the supervision, research design, data analysis, interpretation and writing and editing.

## CONFLICT OF INTEREST

The authors declare no conflict of interest.

## ETHICS STATEMENT

Ethical approval was obtained from the university's HREC—Humanities Committee in October 2020 (HS‐20‐48‐Seery‐Bramham).

## Supporting information

Supplementary information.

## Data Availability

The data set is available for appropriate review and use by contacting the corresponding author.

## References

[1] Agnew‐Blais JC , Polanczyk GV , Danese A , Wertz J , Moffitt TE , Arseneault L . Evaluation of the persistence, remission, and emergence of attention‐deficit/hyperactivity disorder in young adulthood. JAMA Psychiatry. 2016;73(7):713‐720. 10.1001/jamapsychiatry.2016.0465 27192174 PMC5475268

[2] Barbaresi WJ , Colligan RC , Weaver AL , Voigt RG , Killian JM , Katusic SK . Mortality, ADHD, and psychosocial adversity in adults with childhood ADHD: a prospective study. Pediatrics. 2013;131(4):637‐644. 10.1542/peds.2012-2354 23460687 PMC3821174

[3] Knouse LE , Cooper‐Vince C , Sprich S , Safren SA . Recent developments in the psychosocial treatment of adult ADHD. Expert Rev Neurother. 2008;8(10):1537‐1548. 10.1586/14737175.8.10.1537 18928346 PMC2628311

[4] Bramham J , Young S , Bickerdike A , Spain D , McCartan D , Xenitidis K . Evaluation of group cognitive behavioral therapy for adults with ADHD. J Atten Disord. 2009;12(5):434‐441. 10.1177/1087054708314596 18310557

[5] Hirvikoski T , Lindström T , Carlsson J , Waaler E , Jokinen J , Bölte S . Psychoeducational groups for adults with ADHD and their significant others (PEGASUS): a pragmatic multicenter and randomized controlled trial. Eur Psychiatry. 2017;44:141‐152. 10.1016/j.eurpsy.2017.04.005 28641216

[6] Hoxhaj E , Sadohara C , Borel P , et al. Mindfulness vs. psychoeducation in adult ADHD: a randomized controlled trial. Eur Arch Psychiatry Clin Neurosci. 2018;268(4):321‐335. 10.1007/s00406-018-0868-4 29356899

[7] Bonati M , Cartabia M , Zanetti M , Lombardy ADHD G . Waiting times for diagnosis of attention‐deficit hyperactivity disorder in children and adolescents referred to Italian ADHD centers must be reduced. BMC Health Serv Res. 2019;19(1):673. 10.1186/s12913-019-4524-0 31533711 PMC6751639

[8] Casey T , Johnson C , Love D . Adult attention deficit hyperactivity disorder clinic: an interprofessional collaboration. J Am Pharm Assoc. 2020;60(suppl 5):S113‐S117. 10.1016/j.japh.2020.03.020 32616446

[9] Cheung KK , Wong IC , Ip P , et al. Experiences of adolescents and young adults with ADHD in Hong Kong: treatment services and clinical management. BMC Psychiatry. 2015;15:95. 10.1186/s12888-015-0478-x 25927845 PMC4459071

[10] Aguilera A . Digital technology and mental health interventions: opportunities and challenges. Arbor. 2015;191(771):a210. 10.3989/arbor.2015.771n1012

[11] Khan K , Hall CL , Davies EB , Hollis C , Glazebrook C . The effectiveness of web‐based interventions delivered to children and young people with neurodevelopmental disorders: systematic review and meta‐analysis. J Med Internet Res. 2019;21(11):e13478. 10.2196/13478 31682573 PMC6858614

[12] Linardon J , Shatte A , Messer M , Firth J , Fuller‐Tyszkiewicz M . E‐mental health interventions for the treatment and prevention of eating disorders: an updated systematic review and meta‐analysis. J Consult Clin Psychol. 2020;88(11):994‐1007. 10.1037/ccp0000575 32852971

[13] Patel S , Akhtar A , Malins S , et al. The acceptability and usability of digital health interventions for adults with depression, anxiety, and somatoform disorders: qualitative systematic review and meta‐synthesis. J Med Internet Res. 2020;22(7):e16228. 10.2196/16228 32628116 PMC7381032

[14] National Institute for Health and Care Excellence. *Attention deficit hyperactivity disoder: diagnosis and management*. 2018. Accessed September 21, 2021. https://www.nice.org.uk/guidance/ng87 29634174

[15] Powell L , Parker J , Harpin V , Mawson S . Guideline development for technological interventions for children and young people to self‐manage attention deficit hyperactivity disorder: realist evaluation. J Med Internet Res. 2019;21(4):e12831. 10.2196/12831 30942692 PMC6468334

[16] Young S , Adamo N , Ásgeirsdóttir BB , et al. Females with ADHD: an expert consensus statement taking a lifespan approach providing guidance for the identification and treatment of attention‐deficit/hyperactivity disorder in girls and women. BMC Psychiatry. 2020;20(1):404. 10.1186/s12888-020-02707-9 32787804 PMC7422602

[17] Moëll B , Kollberg L , Nasri B , Lindefors N , Kaldo V . Living SMART—a randomized controlled trial of a guided online course teaching adults with ADHD or sub‐clinical ADHD to use smartphones to structure their everyday life. Internet Interv. 2015;2(1):24‐31. 10.1016/j.invent.2014.11.004

[18] Jang S , Kim JJ , Kim SJ , Hong J , Kim S , Kim E . Mobile app‐based chatbot to deliver cognitive behavioral therapy and psychoeducation for adults with attention deficit: a development and feasibility/usability study. Int J Med Inform. 2021;150:104440. 10.1016/j.ijmedinf.2021.104440 33799055

[19] Powell L , Parker J , Harpin V . What is the level of evidence for the use of currently available technologies in facilitating the self‐management of difficulties associated with ADHD in children and young people? A systematic review. Eur Child Adolesc Psychiatry. 2018;27(11):1391‐1412. 10.1007/s00787-017-1092-x 29222634

[20] Kollins SH , DeLoss DJ , Cañadas E , et al. A novel digital intervention for actively reducing severity of paediatric ADHD (STARS‐ADHD): a randomised controlled trial. Lancet Digit Health. 2020;2(4):e168‐e178. 10.1016/S2589-7500(20)30017-0 33334505

[21] Schuck S , Emmerson N , Ziv H , et al. Designing an iPad app to monitor and improve classroom behavior for children with ADHD: iSelfControl feasibility and pilot studies. PLoS One. 2016;11(10):e0164229. 10.1371/journal.pone.0164229 27741257 PMC5065230

[22] Canela C , Buadze A , Dube A , Eich D , Liebrenz M . Skills and compensation strategies in adult ADHD—a qualitative study. PLoS One. 2017;12(9):e0184964. 10.1371/journal.pone.0184964 28953946 PMC5617155

[23] Simons L , Valentine AZ , Falconer CJ , et al. Developing mHealth remote monitoring technology for attention deficit hyperactivity disorder: a qualitative study eliciting user priorities and needs. JMIR Mhealth Uhealth. 2016;4(1):e5009. 10.2196/mhealth.5009 PMC4823590 27009498

[24] Flobak E , Nordby ES , Guribye F , Kenter R , Nordgreen T , Lundervold AJ . Designing videos with and for adults with ADHD for an online intervention: participatory design study and thematic analysis of evaluation. JMIR Ment Health. 2021;8(9):e30292. 10.2196/30292 34519666 PMC8479608

[25] Păsărelu CR , Andersson G , Dobrean A . Attention‐deficit/hyperactivity disorder mobile apps: a systematic review. Int J Med Inform. 2020;138:104133. 10.1016/j.ijmedinf.2020.104133 32283479

[26] Hidalgo‐Mazzei D , Reinares M , Mateu A , et al. OpenSIMPLe: a real‐world implementation feasibility study of a smartphone‐based psychoeducation programme for bipolar disorder. J Affect Disord. 2018;241:436‐445. 10.1016/j.jad.2018.08.048 30145515

[27] Powell L , Parker J , Harpin V . ADHD: is there an app for that? A suitability assessment of apps for the parents of children and young people with ADHD. JMIR Mhealth Uhealth. 2017;5(10):e7941. 10.2196/mhealth.7941 PMC5660294 29030325

[28] Spiel K , Hornecker E , Williams RM , Good J . ADHD and technology research—investigated by Neurodivergent Readers Association for Computing Machinery. Paper presented at: CHI '22: CHI Conference on Human Factors in Computing Systems; 2022. pp. 1‐21. 10.1145/3491102.3517592

[29] Bevan Jones R , Stallard P , Agha SS , et al. Practitioner review: co‐design of digital mental health technologies with children and young people. J Child Psychol Psychiatry. 2020;61(8):928‐940. 10.1111/jcpp.13258 32572961 PMC7611975

[30] Wykes T , Brown M . Over promised, over‐sold and underperforming?—e‐health in mental health. J Ment Health. 2016;25(1):1‐4. 10.3109/09638237.2015.1124406 26732733 PMC4776695

[31] Boulkedid R , Abdoul H , Loustau M , Sibony O , Alberti C . Using and reporting the delphi method for selecting healthcare quality indicators: a systematic review. PLoS One. 2011;6(6):e20476. 10.1371/journal.pone.0020476 21694759 PMC3111406

[32] Niederberger M , Spranger J . Delphi technique in health sciences: a map. Front Public Health. 2020;8:457. 33072683 10.3389/fpubh.2020.00457 PMC7536299

[33] Kelly CM , Jorm AF , Kitchener BA , Langlands RL . Development of mental health first aid guidelines for suicidal ideation and behaviour: a Delphi study. BMC Psychiatry. 2008;8(1):17. 10.1186/1471-244X-8-17 18366657 PMC2324091

[34] Lakeman R . Mental health recovery competencies for mental health workers: a Delphi study. J Ment Health. 2010;19(1):62‐74. 10.3109/09638230903469194 20380499

[35] McGinn CA , Gagnon MP , Shaw N , et al. Users' perspectives of key factors to implementing electronic health records in Canada: a Delphi study. BMC Med Inform Decis Mak. 2012;12(1):105. 10.1186/1472-6947-12-105 22967231 PMC3470948

[36] Silva MT , Silva EN , , da Barreto JOM . Rapid response in health technology assessment: a Delphi study for a Brazilian guideline. BMC Med Res Methodol. 2018;18(1):51. 10.1186/s12874-018-0512-z 29884121 PMC5994001

[37] van Rijssen LB , Gerritsen A , Henselmans I , et al. Core set of patient‐reported outcomes in pancreatic cancer (COPRAC): an International Delphi Study among patients and health care providers. Ann Surg. 2019;270(1):158‐164. 10.1097/SLA.0000000000002633 29261524

[38] Ahmed R , McCaffery KJ , Aslani P . Development and validation of a question prompt list for parents of children with attention‐deficit/hyperactivity disorder: a Delphi study. Health Expect. 2016;19(2):234‐252. 10.1111/hex.12341 25597620 PMC5055277

[39] Fuermaier ABM , Fricke JA , de Vries SM , Tucha L , Tucha O . Neuropsychological assessment of adults with adhd: a Delphi consensus study. Appl Neuropsychol Adult. 2019;26(4):340‐354. 10.1080/23279095.2018.1429441 29424567

[40] Hervás A , de Santos T , Quintero J , et al. Delphi consensus on attention deficit hyperactivity disorder (ADHD): evaluation by a panel of experts. Actas Esp Psiquiatr. 2016;44(6):231‐243. 27906414

[41] Brandl L , Cabrita M , Brodbeck J , Heylen D , van Velsen L . Consulting the oracle: a Delphi study for determining parameters for a mental health user profile and personalization strategy for an online service to aid grieving older adults. Internet Interv. 2022;28:100534. 10.1016/j.invent.2022.100534 35462943 PMC9019256

[42] Byrne R , Morrison AP . Service users' priorities. and preferences for treatment of psychosis: a user‐led Delphi study. Psychiatr Serv. 2014;65(9):1167‐1169. 10.1176/appi.ps.201300289 24933125

[43] Novais T , Mouchoux C , Kossovsky M , et al. Neurocognitive disorders: what are the prioritized caregiver needs? A consensus obtained by the Delphi method. BMC Health Serv Res. 2018;18:1016. 10.1186/s12913-018-3826-y 30594202 PMC6311000

[44] Benninger E , Savahl S . The children's Delphi: considerations for developing a programme for promoting children's self‐concept and well‐being. Child Fam Soc Work. 2017;22(2):1094‐1103. 10.1111/cfs.12329

[45] Quayle E , Cariola L . Management of non‐consensually shared youth‐produced sexual images: a Delphi study with adolescents as experts. Child Abuse Negl. 2019;95:104064. 10.1016/j.chiabu.2019.104064 31279956

[46] Higgins JM , Arnold SR , Weise J , Pellicano E , Trollor JN . Defining autistic burnout through experts by lived experience: grounded Delphi method investigating #AutisticBurnout. Autism. 2021;25(8):2356‐2369. 10.1177/13623613211019858 34088219

[47] Benevides TW , Shore SM , Palmer K , et al. Listening to the autistic voice: mental health priorities to guide research and practice in autism from a stakeholder‐driven project. Autism. 2020;24(4):822‐833. 10.1177/1362361320908410 32429818 PMC7787673

[48] Spiel K , Gerling K . The purpose of play: how HCI games research fails neurodivergent populations. ACM Trans Comput Hum Interact. 2021;28(2):1‐11. 10.1145/3432245

[49] Powell C . The Delphi technique: myths and realities. J Adv Nurs. 2003;41(4):376‐382. 10.1046/j.1365-2648.2003.02537.x 12581103

[50] Langbecker D , Janda M , Yates P . Development and piloting of a brain tumour‐specific question prompt list. Eur J Cancer Care. 2012;21(4):517‐526. 10.1111/j.1365-2354.2012.01328.x 22309311

[51] Braun V , Clarke V . Using thematic analysis in psychology. Qual Res Psychol. 2006;3(2):77‐101. 10.1191/1478088706qp063oa

[52] Scheibe M , Skutsch M , Schofer J . Experiments in Delphi methodology. In: Linstone HA , Turoff M , eds. The Delphi Method: Techniques and Applications. Addison‐Wesley.

[53] Raskin MS . The Delphi study in field instruction revisited: expert consensus on issues and research priorities. J Soc Work Educ. 1994;30(1):75‐89. 10.1080/10437797.1994.10672215

[54] Rayens MK , Hahn EJ . Building consensus using the policy Delphi method. Policy Polit Nurs Pract. 2000;1(4):308‐315. 10.1177/152715440000100409

[55] Aoki Y , Furuno T , Watanabe K , Kayama M . The experiences of receiving a diagnosis of attention deficit hyperactivity disorder during adulthood in Japan: a qualitative study. BMC Psychiatry. 2020;20:1‐8. 10.1186/s12888-020-02774-y 32677922 PMC7366299

[56] Barrington H , Young B , Williamson PR . Patient participation in Delphi surveys to develop core outcome sets: systematic review. BMJ Open. 2021;11(9):e051066. 10.1136/bmjopen-2021-051066 PMC8413947 34475183

[57] Schmalz U , Spinler S , Ringbeck J . Lessons learned from a two‐round Delphi‐based scenario study. MethodsX. 2021;8:101179. 10.1016/j.mex.2020.101179 33365260 PMC7749424

[58] Gargon E , Crew R , Burnside G , Williamson PR . Higher number of items associated with significantly lower response rates in COS Delphi surveys. J Clin Epidemiol. 2019;108:110‐120. 10.1016/j.jclinepi.2018.12.010 30557677 PMC6438267

[59] Chandrashekar P . Do mental health mobile apps work: evidence and recommendations for designing high‐efficacy mental health mobile apps. Mhealth. 2018;4:6. 10.21037/mhealth.2018.03.02 29682510 PMC5897664

[60] Lattie EG , Stiles‐Shields C , Graham AK . An overview of and recommendations for more accessible digital mental health services. Nat Rev Psychol. 2022;1(2):87‐100. 10.1038/s44159-021-00003-1 PMC10956902 38515434

[61] Price M , Yuen EK , Goetter EM , et al. mHealth: a mechanism to deliver more accessible, more effective mental health care. Clin Psychol Psychother. 2014;21(5):427‐436. 10.1002/cpp.1855 23918764 PMC3926903

[62] Pywell J , Vijaykumar S , Dodd A , Coventry L . Barriers to older adults' uptake of mobile‐based mental health interventions. Digit Health. 2020;6:2055207620905422. 10.1177/2055207620905422 32110429 PMC7016304

[63] Schröder J , Berger T , Westermann S , Klein JP , Moritz S . Internet interventions for depression: new developments. Dialogues Clin Neurosci. 2016;18(2):203‐212. 27489460 10.31887/DCNS.2016.18.2/jschroeder PMC4969707

[64] de la Torre‐Díez I , López‐Coronado M , Vaca C , Aguado JS , de Castro C . Cost‐utility and cost‐effectiveness studies of telemedicine, electronic, and mobile health systems in the literature: a systematic review. Telemed J E Health. 2015;21(2):81‐85. 10.1089/tmj.2014.0053 25474190 PMC4312789

[65] Jiang X , Ming WK , You JH . The cost‐effectiveness of digital health interventions on the management of cardiovascular diseases: systematic review. J Med Internet Res. 2019;21(6):e13166. 10.2196/13166 31210136 PMC6601257

[66] Health Service Executive . ADHD in adults NCP model of care. 2021. https://www.hse.ie/eng/about/who/cspd/ncps/mental-health/adhd/adhd-in-adults-ncp-model-of-care/

[67] Cogley C , D'Alton P , Nolan M , Smith E . “You were lying in limbo and you knew nothing”: a thematic analysis of the information needs of spinal cord injured patients and family members in acute care. Disabil Rehabil . 2021;1‐11. 10.1080/09638288.2021.1970259 34465272

[68] ADHD Europe . *Diagnosis and Treatment of ADHD in Europe*. ADHD Europe; 2020. Accessed April 4, 2021. https://adhdeurope.eu/wp-content/uploads/2021/04/Survey-2020-FINAL.pdf

[69] Young S , Asherson P , Lloyd T , et al. Failure of healthcare provision for attention‐deficit/hyperactivity disorder in the United Kingdom: a consensus statement. Front Psychiatry. 2021;12:649399. 10.3389/fpsyt.2021.649399 33815178 PMC8017218

[70] Carr‐Fanning K , Mc Guckin C . The powerless or the empowered? Stakeholders' experiences of diagnosis and treatment for attention‐deficit hyperactivity disorder in Ireland. Ir J Psychol Med. 2018;35(3):203‐212. 10.1017/ipm.2018.13 30124187

[71] Hemingway SL , Cameron EC , Jacquin KM . College students who believe they have ADHD report more neuropsychological deficits than non‐ADHD peers. J Am Coll Health . 2021;1‐8. 10.1080/07448481.2021.1963737 34448674

[72] Nasa P , Jain R , Juneja D . Delphi methodology in healthcare research: how to decide its appropriateness. World J Methodol. 2021;11(4):116‐129. 10.5662/wjm.v11.i4.116 34322364 PMC8299905

[73] Beiderbeck D , Frevel N , von der Gracht HA , Schmidt SL , Schweitzer VM . Preparing, conducting, and analyzing Delphi surveys: cross‐disciplinary practices, new directions, and advancements. MethodsX. 2021;8:101401. 10.1016/j.mex.2021.101401 34430297 PMC8374446

