# Supplementary material for: What adults with ADHD want to know: A Delphi consensus study on the psychoeducational needs of experts by experience
Source: Health Expect. 2022 Aug 23;25(5):2593–602. doi: 10.1111/hex.13592 (PMC9615057; doi:10.1111/hex.13592)
Supplement: Supplementary file 1 — Supplementary information. [file HEX-25--s001.docx]

**Topics in round one**

***Background information on adult ADHD***

1. The history of knowledge on ADHD
2. What is the nature of ADHD
3. The prevalence of ADHD
4. Comparing childhood and adult ADHD
5. Executive functioning
6. Core types of ADHD
7. Using ADHD to your advantage
8. Symptom severity
9. Impulsivity
10. Hyperactivity
11. Inattention
12. Mood/emotional regulation
13. Physical symptoms
14. Symptom manifestation across different situations
15. Typical manifestations of ADHD
16. Hereditary and biological factors
17. Psychosocial factors
18. Gender ratio
19. How ADHD affects men and women differently

***A Diagnosis of ADHD***

1. What is a diagnosis of ADHD
2. Who can give a diagnosis
3. What is the process of getting a diagnosis
4. What should a thorough assessment look like
5. Diagnostic interviews
6. Standardized behaviour rating scales
7. Interviews of loved ones
8. What to consider when getting a diagnosis
9. How to know if you need an evaluation for ADHD
10. Assessment of social skills
11. The impact of undiagnosed ADHD
12. ADHD Stigma
13. Feelings of low self-esteem, embarrassment, guilt or blame
14. Deciding to disclose a diagnosis
15. Disclosing a diagnosis at work/university
16. What are comorbidities
17. What are the most common comorbidities
18. The role of gender and comorbidities
19. Emotions and mood swings
20. Anxiety
21. ADHD & self-harm
22. Addiction
23. Sleep difficulties and disorders
24. Physical conditions (eg. asthma or epilepsy)
25. Weight management
26. Nutrition
27. Exercise
28. Why might someone with ADHD have distress?
29. Distinguishing ADHD from other disorders
30. Practicing self-care
31. What are specific learning disabilities (eg. dyslexia, autism spectrum disorder, dyspraxia)
32. Prevalence of specific learning disabilities
33. Education and specific learning disabilities

***Treatment***

1. Transition from CAMHS to adult services
2. Accessing services
3. Questions for your practitioner
4. Finding a professional
5. Necessity of integrated approaches
6. Available treatment options
7. When to seek treatment
8. How can treatment help
9. Common components of treatment plans
10. Initial steps in treatment
11. Effectiveness of medication
12. Physical assessment for medication
13. Types of medication prescribed
14. Side effects of medication
15. Process of going on medication (dosage, prescription, monitoring)
16. Annual reviews of medication
17. Challenges of medical treatment for someone with ADHD
18. When to start a trial of medication
19. Once a day medication VS short acting medication
20. Trial and error with medication (trying medication, what to do if the medication doesn’t suit)
21. Medication and pregnancy
22. Treating co-existing conditions (anxiety, depression) and medication
23. Medication management
24. Psychoeducation
25. Therapies (eg. Cognitive Behavioural Therapy, Dialectical Behavioural Therapy)
26. Relaxation training and stress management
27. Parent training
28. Group therapy
29. Behavioural coaching, job coach, family education
30. Support from organisations
31. Occupational therapy
32. Speech therapy
33. ADHD coaches
34. Adult peer supports
35. Recognising ‘snake oil’ treatments

***Living with ADHD***

*Relationships*

1. Parenting
2. Impact of ADHD on relationships
3. How education can help relationships
4. ADHD & Social skills
5. Impact of ADHD symptoms on social situations

*Laws and rights*

1. Educational support and rights
2. Employer responsibility
3. Disabilities and confidentiality
4. Job discrimination
5. Disability acts
6. Finding legal advocates
7. Terms to know
8. Necessary documents for legal purposes

*Occupational/Educational settings*

1. Challenges at work
2. Choosing a career
3. Requesting accommodations
4. Working from home
5. Managing work related challenges
6. Staying focused while studying
7. Managing procrastination
8. Time and task management
9. Improving organisation
10. The effect of ADHD on academic and career success

*Finances*

1. Managing finances
2. How symptoms can affect finances
3. Saving money
4. Money management schedules

*Driving*

1. Notifying the Road Safety Authority
2. Risks of driving with ADHD/distracted driving
3. Safe driving tips for ADHD
4. Driving insurance

**Topics suggested by participants**

1. Developing routines
2. Effects on hygiene
3. Connection to other phone applications
4. To-do list
5. Reminders in taking medication and for hydrating
6. Comparing ADHD functioning with more typical functioning as a way of recognising ADHD
7. Stigmatising responses to ADHD
8. Explaining the legitimacy of ADHD to others
9. Gender disparity in diagnosis
10. Masking
11. ADHD and Autism
12. Lesser known symptoms
13. Disassociation
14. Sensory processing
15. Receiving a diagnosis later in life
16. Finding the right practitioner
17. Managing ADHD without medication
18. When people seek ADHD medication without a clinical diagnosis of ADHD
19. What managed ADHD looks like
20. Task prioritisation
21. Risk management
22. Pros and cons of hyperfocus
23. Disconnecting from hyperfocus
24. ADHD & sports/athletes
25. Self-help books and Youtube channels
26. Working in careers that are challenging
27. Support from social media
28. Finding/locating schools’ and colleges’ policies
29. Parenting a child with ADHD as an adult with ADHD
30. Spotting ADHD in young people
31. What positive groundwork can be laid down by adults with ADHD for children they suspect might have ADHD
32. ADHD and menopause
33. ADHD and sexual intimacy
34. Recognising abusive romantic relationships
35. Loving and accepting your ADHD
36. What ADHD looks like in the brain
37. Up to date research
